# Glycosylation Tunes Neuroserpin Physiological and Pathological Properties

**DOI:** 10.3390/ijms21093235

**Published:** 2020-05-03

**Authors:** Cristina Visentin, Luca Broggini, Benedetta Maria Sala, Rosaria Russo, Alberto Barbiroli, Carlo Santambrogio, Simona Nonnis, Anatoly Dubnovitsky, Martino Bolognesi, Elena Miranda, Adnane Achour, Stefano Ricagno

**Affiliations:** 1 Dipartimento di Bioscienze, Università degli Studi di Milano, Via Celoria, 26, 20133 Milan, Italy; cristina.visentin@unimi.it (C.V.); luca.broggini@unimi.it (L.B.); bmsala@kth.se (B.M.S.); martino.bolognesi@unimi.it (M.B.); 2 Science for Life Laboratory, Department of Medicine Solna, Karolinska Institute, and Division of Infectious Diseases, Karolinska University Hospital, Solna, SE-17176 Stockholm, Sweden; adnane.achour@ki.se; 3 Department of Protein Science, School of Engineering Sciences in Chemistry, Biotechnology and Health, AlbaNova University Center, Royal Institute of Technology, SE-10691 Stockholm, Sweden; 4 Dipartimento di Fisiopatologia Medico-Chirurgica e dei Trapianti, Università degli Studi di Milano, Via Fratelli Cervi, 93, 20090 Segrate, Italy; rosaria.russo@unimi.it; 5 Dipartimento di Scienze per gli Alimenti, la Nutrizione e l′Ambiente, Università degli Studi di Milano, Via Celoria, 2, 20133 Milan, Italy; alberto.barbiroli@unimi.it; 6 Dipartimento di Biotecnologie e Bioscienze, Università degli Studi di Milano-Bicocca, Piazza dell’Ateneo Nuovo, 1, 20126 Milan, Italy; carlo.santambrogio@unimib.it; 7 Departimento di Medicina Veterinaria, Università degli Studi di Milano, Via dell’Università, 6, 26900 Lodi, Italy; Simona.Nonnis@unimi.it; 8 Science for Life Laboratory, Department of Medicine Solna, Karolinska Institutet, and Division of Rheumatology, Karolinska University Hospital, Solna, SE-17176 Stockholm, Sweden; anatoly.dubnovitsky@ki.se; 9 Dipartimento di Biologia e Biotecnologie ‘Charles Darwin’, and Istituto Pasteur - Fondazione Cenci-Bolognetti, Sapienza Università di Roma, Piazzale Aldo Moro, 5, 00185 Rome, Italy; mariaelena.mirandabanos@uniroma1.it

**Keywords:** neuroserpin, protein polymerisation, glycosylation

## Abstract

Neuroserpin (NS) is a member of the serine protease inhibitors superfamily. Specific point mutations are responsible for its accumulation in the endoplasmic reticulum of neurons that leads to a pathological condition named familial encephalopathy with neuroserpin inclusion bodies (FENIB). Wild-type NS presents two N-glycosylation chains and does not form polymers in vivo, while non-glycosylated NS causes aberrant polymer accumulation in cell models. To date, all in vitro studies have been conducted on bacterially expressed NS, de facto neglecting the role of glycosylation in the biochemical properties of NS. Here, we report the expression and purification of human glycosylated NS (gNS) using a novel eukaryotic expression system, LEXSY. Our results confirm the correct N-glycosylation of wild-type gNS. The fold and stability of gNS are not altered compared to bacterially expressed NS, as demonstrated by the circular dichroism and intrinsic tryptophan fluorescence assays. Intriguingly, gNS displays a remarkably reduced polymerisation propensity compared to non-glycosylated NS, in keeping with what was previously observed for wild-type NS in vivo and in cell models. Thus, our results support the relevance of gNS as a new in vitro tool to study the molecular bases of FENIB.

## 1. Introduction

Neuroserpin (NS) is a human protein mainly expressed in the nervous system [1]. This protein is ascribed to the serine protease inhibitor (serpin) superfamily with which it shares the conserved serpin fold and mechanism of action [1,2]. Even though its physiological roles are not completely elucidated, NS activity is involved in memory, learning, and both synaptic and neurovascular compartment plasticity [3,4,5]. NS, as any member of the serpin superfamily, is characterised by the presence of a long and flexible loop, named the reactive centre loop (RCL), essential for its physiological function [6,7]. The RCL is recognized by the tissue plasminogen activator (tPA), the target protease [8]. Upon binding to NS, tPA hydrolyses the RCL loop at position Arg362 with the concomitant formation of acyl complex NS-tPA. The RCL cleavage triggers a major structural rearrangement in which the N-terminal portion of the cleaved RCL is inserted into the central ß-sheet A between strands 3A and 5A [9]. Typically, in serpins, such conformational change causes the disruption of the protease active site and prevents the hydrolysis of the acyl-complex, rendering this covalent complex extremely stable over time [10]. During the conformational change from the native to the RCL-cleaved form, a consistent stabilization of the serpin molecule takes place, yielding a cleaved form that is hyper-stable [11]. However, opposite to other serpin-protease pairs, the NS-tPA complex is short-living and rapidly dissociates at physiological pH, releasing free cleaved NS and active tPA [6,12,13]. Another serpin inhibitor of tPA, plasminogen activator inhibitor-1 (PAI1), is instead forming a long-living acyl-complex whose dissociation has never been observed [12]. Compared to PAI1, NS discriminates between tPA and uPA (urokinase-type plasminogen activator), and between the single- or double-chain tPA [12]. The pH also plays an important role in the stability of the NS-tPA complex [13]. Lee et al. demonstrated that strands sC1 and sC2 and helices hCD and hE contribute to the recruitment of tPA and to the stabilization of the NS-tPA complex [14]. Monomeric NS can access a third conformation, the latent fold, where the uncleaved RCL is inserted in the β-sheet A, similar to the cleaved conformation [2,11]. Both these NS conformations are extremely stable and unable to load, and consequently to inhibit, tPA. 

NS mutations are responsible for the onset of familial encephalopathy with neuroserpin inclusion bodies (FENIB), a severe and fatal serpinopathy characterized by progressive neurodegeneration. Epilepsy, cognitive impairment and dementia are the main clinical symptoms reported for the patients. FENIB is a rare and autosomally dominant genetic disorder for which the age of onset and the severity of clinical manifestations are strictly correlated to the specific mutation carried by the patient [15]. There are six known point mutations related to FENIB: S49P, S52R, H338R, G392E, G392R, and L47P [16,17,18,19]. Mutated NS undergoes polymerization and deposits within the endoplasmic reticulum of neurons, where it accumulates as inclusion bodies [17,20] causing a poorly characterized neuronal toxicity that involves oxidative stress and apoptosis [21]. The structural features of polymeric NS are not completely understood yet. In all the polymerisation models reported for the prototypical serpin alpha-1 antitrypsin, the RCL loop exerts a crucial role and is thought to be inserted in β-sheet A of the neighbouring monomers as part of the intermolecular link [22,23], or intramolecularly to provide flexibility for the domain swap that links monomers into polymeric chains [23]. 

The monomeric and polymeric NS conformers can be discriminated using specific structural signatures in different spectroscopic techniques: the circular dichroism (CD) spectra of latent and polymeric NS show a more intense signal compared to native NS [24,25], and Noto et al. reported the possibility to profile each conformer using protein intrinsic fluorescence, i.e., the emission spectra of tryptophan or tyrosine residues within NS [25].

As many other secreted proteins, human NS is glycosylated in vivo and presents two N-linked glycosylation chains on asparagine residues N157 and N321. A third aberrant glycosylation chain added in N401 has been observed for the G392E pathological NS mutant and causes abundant polymer accumulation within the endoplasmic reticulum in cell models of FENIB [15,26,27,28,29,30]. The presence of the post-translational modifications is known to play an important role in the NS protein quality control [29] and stability [29,30]. In particular, Schipanski et al. [29] and Moriconi et al. [30] reported that the glycosylation plays a pivotal role in keeping NS in its physiological monomeric state in HEK and COS-7 cells. When the physiologic glycosylation pattern is artificially impaired by the specific mutation of N157 and N321 residues, the behaviour of wild-type NS is reminiscent of the pathological FENIB mutants, and the accumulation of NS polymers was observed in the endoplasmic reticulum [30]. The effect was additive and the abrogation of both glycosylation sites caused an even more pronounced accumulation of NS polymers [30]. 

To date, only rat NS has been expressed glycosylated [31]; indeed, all the in vitro biochemical and biophysical studies on human NS have been conducted using recombinant protein purified from *E. coli* [8]. Curiously, in contrast to the studies conducted in vivo and in cell cultures models, purified NS efficiently polymerises in vitro even after short incubation times and at temperatures only slightly higher than the physiological 37 °C [24,25,32]. This suggests that, although technically convenient, bacterially expressed human NS does not reproduce the behaviour of this protein as observed in more physiologic contexts. These considerations highlighted the need to assess the role of the N-glycosylation in the molecular properties of the human wild-type NS in vitro. Thus, in order to shed light on this conundrum, we report here for the first time the expression, purification and characterisation of recombinant N-glycosylated human NS (gNS) produced using LEXSY^®^ (*Leishmania tarentolae* expression system), an eukaryotic expression system based on *L. tarentolae* cells [33]. N-glycosylation by *Leishmania* spp. is more equivalent to the mammalian counterpart compared to other model organisms, e.g., insect cells or fungi [33]. For this reason, the use of LEXSY is particularly suitable for the expression of human glycosylated protein. 

The presence of the correct glycosylation pattern and the conformational and biophysical properties of gNS were assessed in comparison with non-glycosylated bacterially purified NS, confirming the correct fold of the glycosylated variant (gNS). A marked reduction was observed in the heat-induced polymerisation propensity of gNS compared to NS. Finally, gNS displays a slightly increased efficiency in inhibiting tPA activity in vitro. Taken together, here, we produced a glycosylated version of NS that shares all molecular properties of native NS but, importantly, it better recapitulates NS polymerisation propensity, as observed in vivo and in cell models. Thus, gNS should be considered a valuable new in vitro tool to study NS polymerisation and its inhibition. 

## 2. Results and Discussion

### 2.1. Expression and Purification of Glycosylated NS

**Figure 1:**
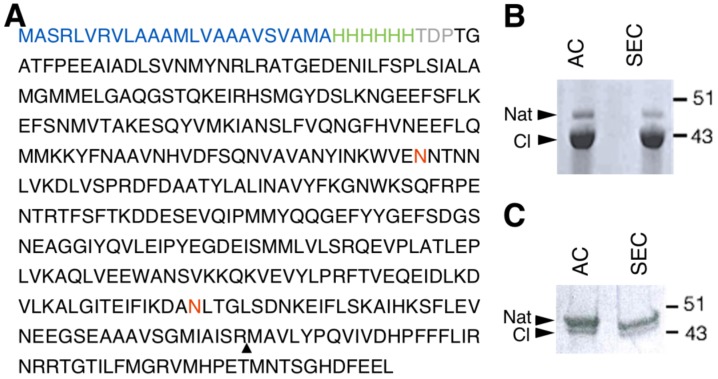
Human glycosylated NS (gNS) expression and purification in LEXSY. (**A**) Primary sequence of the construct used for gNS expression in LEXSY (UniProtKB - Q99574). The signal peptide sequence is colored in blue, the HisTag is green and the spacer in grey. The neuroserpin (NS) sequence is black, the glycosylation sites are in red and the triangle highlights the protease cleavage site. (**B**) SDS-PAGE analysis of gNS purification after AC and SEC chromatographic steps according to the protocol shown in Figure S1A. (**C**) SDS-PAGE analysis of optimized gNS purification after the AC and SEC chromatographic steps (see also Figure S1B). Abbreviations: Nat: native; CL: cleaved; AC: affinity chromatography; SEC: size exclusion chromatography.

In order to successfully express human NS in the LEXSY system, a *Leishmania*-optimised sequence of the NS gene was cloned into pLEXSY-sat2, an integrative plasmid for constitutive expression. The NS gene was cloned in frame with a 6-His tag engineered at the N-*terminus*. To improve the amount of secreted protein, the commercial secretion signal peptide was substituted with the modified sequence of the SP5 signal peptide (Figure 1A) [34]. Once stable expressing clones were obtained, protein expression was carried out for 60 h at 26 °C. NS expressed in LEXSY (thereafter named gNS) was purified from the growth medium after removing intact cells and cellular debris. Two steps of chromatography were performed to isolate the gNS monomer: an initial Ni-NTA affinity chromatography (AC) was followed by a size exclusion chromatography step (SEC) (Figure S1A). About 7 mg of highly pure monomeric gNS was obtained per litre of LEXSY culture, but, undesirably, nearly 80% of the purified protein was in the cleaved form (Figure 1B). The addition of cocktails of protease inhibitors in the medium did not ameliorate the proteolytic pattern (data not shown). 

In vitro purified NS displayed a highly reproducible tendency to autoproteolysis over time, which is accelerated upon temperature increase (Figure S2). Thus, we hypothesise that the prolonged incubation of NS at 26 °C in LEXSY medium was responsible for this phenomenon. In order to minimize NS cleavage, the expression protocol was modified: after 60 h of culture, cells were collected, resuspended into fresh medium and incubated 16 h at 18 °C (Figure S1B). Next, purification steps were performed as reported above. The purification of gNS from such 16 h of growth resulted in a significantly increased amount of uncleaved native NS; however, the yield was reduced to 2 mg per litre of culture and a minor residual fraction of cleaved gNS was still present (Figure 1C). 

### 2.2. gNS Is Properly Glycosylated 

The presence of appropriate glycosylation on LEXSY-expressed gNS was verified using several complementary techniques. First, the migration of gNS in denaturing polyacrylamide gel electrophoresis (SDS-PAGE) was delayed compared to NS (Figure 6D). The increment of molecular mass was in agreement with the presence of N-glycan chains. 

**Figure 2:**
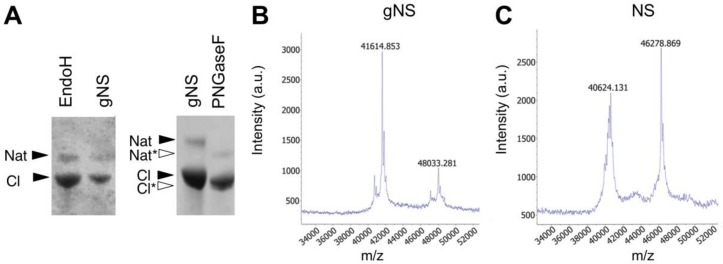
Assessment of N-glycosylation. (**A**) SDS-PAGE analysis of the enzymatic deglycosylation of gNS using EndoH or PNGaseF deglycosylases. All samples are a mixture of native and cleaved forms. Black and white arrows refer to lane 1 (gNS) and lane 2 (PNGaseF), respectively. (**B**,**C**) Matrix-assisted laser desorption ionisation-time of flight (MALDI-TOF) MS spectra of NS (**B**) and gNS. Abbreviations: Nat: native; Nat*: deglycosylated Nat; Cl: cleaved; Cl*: deglycosylated Cl.

The presence of N-linked glycosylation was thereafter confirmed by enzymatic deglycosylation using peptide-N-glycosidase F (PNGaseF) and endoglycosidase H (EndoH). Both enzymes remove N-linked oligosaccharides, but with different specificities. The first enzyme is an amidase that removes all types of mammalian N-linked glycans, whereas EndoH removes only high-mannose and some hybrid types of N-linked glycans. As reported in Figure 2A, gNS was susceptible only to PNGaseF treatment, resulting in a faster migration in SDS-PAGE, whereas incubation with EndoH caused no reduction in molecular mass. Altogether, these results confirm the presence of N-glycosylation along the secretory pathway and the delivery of gNS with mature glycans to the growth medium. In agreement, previously identified N-glycans from an *L. tarentolae* recombinant glycoprotein consisted mostly of the mammalian complex biantennary and the paucimannose Man3GlcNAc2 structures [33]; such glycans are known to be effectively cleaved by PNGase F but not by Endo H [35].

In order to determine the precise protein molecular mass, matrix-assisted laser desorption ionisation-time of flight (MALDI-TOF) MS analyses under native conditions were performed both on gNS and NS proteins. As shown in Figure 2C, the masses of native and cleaved NS, detected at 46,279 and 40,624 Da, respectively, allow for clearly identifying the presence of the cleavage site at Arg362. Moreover, the masses of the gNS protein were detected at 48,033 Da and 41,615 Da for the native and cleaved protein forms, respectively (Figure 2B). The increment in the molecular mass of gNS compared to its theoretical mass unambiguously confirmed the presence of post-translational modifications in gNS. 

**Figure 3:**
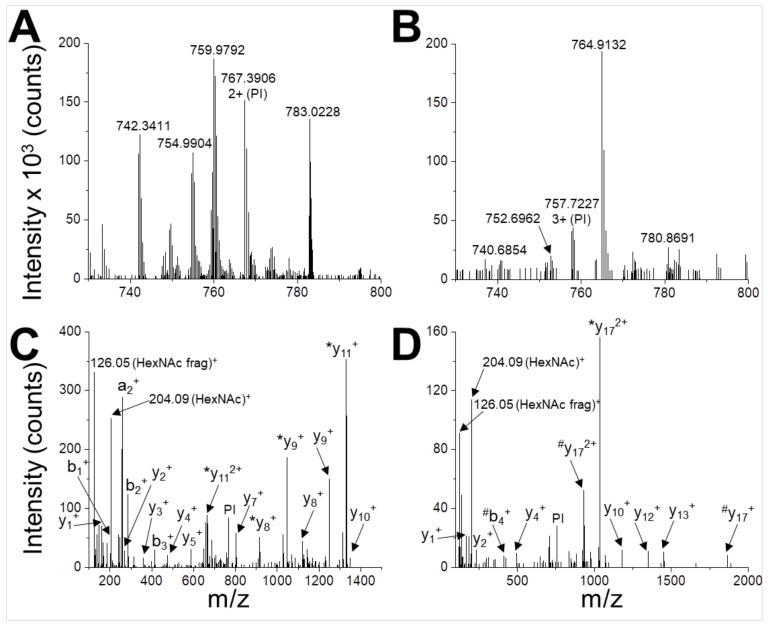
Identification of N-glycosylation sites. UHPLC-MS/MS analysis of glycopeptides WVENNTNNLVK (**A**,**C**) and DANLTGLSDNKEIFLSK (**B**,**D**). The peptides are detected in the MS spectrum as 2+ ion with m/z = 767.3906 (**A**) and 3+ ion with m/z = 757.7227 (**B**). These signals are selected and used as parent ions (PIs) for higher energy collision dissociation (HCD) fragmentation (**C**,**D**). Two glyco-diagnostic peaks are present in the fragmentation spectra (HexNAc, m/z 204.09; HexNAc fragment, m/z 126.05 Da), indicating that PIs are glycopeptides. Identified a-, b- and y-fragment ions are labelled (*, loss of HexNAc during fragmentation; #, loss of HexNAc2 during fragmentation).

In order to map the glycosylation sites, the peptides obtained by the tryptic in-gel digestion of gNS were analysed by UHPLC-MS/MS. The identified gNS peptides, which provided a sequence coverage of 74%, are listed in Table S1 in the Supplementary Materials. Two glycopeptides were detected (Table S1 and Figure 3), corresponding to glycosylation sites at positions N157 and N321. These glycopeptides were not present in a similar analysis performed on gNS deglycosylated by PNGaseF (data not shown). 

Thus, the mass increment observed on gNS compared to NS was due to two occupied N-glycosylation sites. Further experiments would be required to elucidate the corresponding glycan structures.

Overall, these results confirmed that NS produced using the LEXSY expression system presents two N-glycosylations in the sites previously reported [30]. 

### 2.3. Conformational Analysis of gNS

**Figure 4:**
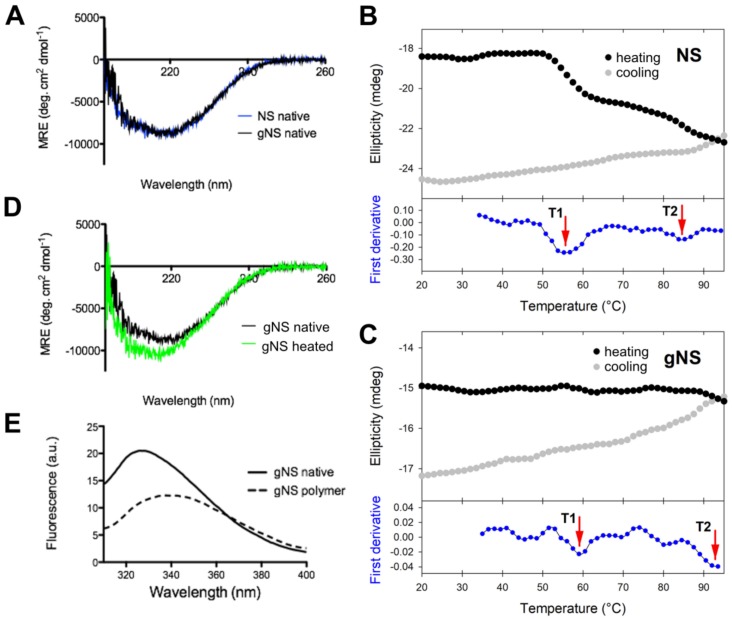
Conformational characterisation of gNS. (**A**) Far-UV circular dichroism (CD) spectra of native gNS and NS recorded at 20 °C. (**B**,**C**) Temperature ramp of NS (**B**) analogously reported in [24] and gNS (**C**) performed recording ellipticity at 216 nm during heating from 20 to 95 °C (black) and cooling down (grey). The first derivative is reported in the bottom (blue) and the two transitions T1 and T2 are highlighted by the red arrows. (**D**) Far-UV CD spectra of gNS recorded at 20 °C before (black) or after (green) heating the sample to 95 °C. (**E**) Intrinsic tryptophan fluorescence of native (solid line) and polymeric (dashed line) gNS recorded at 20 °C. The buffer 20 mM Tris-HCl pH 8, 50 mM KCl was used in all the measurements.

The potential impact of N-glycosylation at residues N157 and N321 on protein structure was evaluated by assessing the secondary structures content of gNS and NS by circular dichroism (CD) analyses. Comparison of the far-UV spectra of gNS and NS is presented in Figure 4A. The two CD spectra perfectly superpose, suggesting that the heterologous expression in LEXSY produces properly folded gNS and that its secondary structure is virtually identical to those of previously characterised NS [6,7]. Even if bacterially expressed NS is not glycosylated, it represents a good reference for NS folding since it was reported that the glycan is not required for the acquirement and maintenance of the serpin fold [30]. Next, the stability and potential conformational changes of gNS were monitored by molar ellipticity at 216 nm, along a temperature ramp from 20 to 95 °C. While bacterial NS undergoes two irreversible transitions at 56.6 and 87.0 °C (Figure 4B in accordance with [24]), the gNS temperature ramp displays two very minor increases (more negative) in CD signal at about 60 and 93 °C (Figure 4C). The conformational changes underlying such signals are irreversible, as the CD signal becomes even more negative when the sample was cooled down (Figure 4C). This typically indicates a gain of secondary structure, connected with latentisation and/or polymerisation reminiscent of what was observed for bacterial NS [24]. The comparison of Far-UV CD spectra recorded before and after the temperature ramp indicate that the latter shows a stronger CD signal (Figure 4D). The more regular structure of the RCL in the latent and polymeric conformers likely accounts for the increased CD signal, as previously reported [24].

It has been reported that intrinsic fluorescence can be informative of NS conformation [25]. For this reason, intrinsic tryptophan fluorescence was recorded to study gNS fold. The spectra of native or SEC-purified polymeric gNS are analysed and compared in Figure 4E. Native gNS presented a main peak at 330 nm, whereas, for the gNS polymers, a red shift and the quenching of the tryptophan signal were detected. These effects observed for polymeric gNS may be ascribed to an increased exposure of tryptophan residues in gNS polymers, similar to what has already been reported in the literature for bacterially expressed NS [7,25,36].

In conclusion, these data indicate that gNS is properly folded and that its overall structure closely resembles the one of bacterially expressed NS.

### 2.4. gNS Inhibits tPA In Vitro

**Figure 5:**
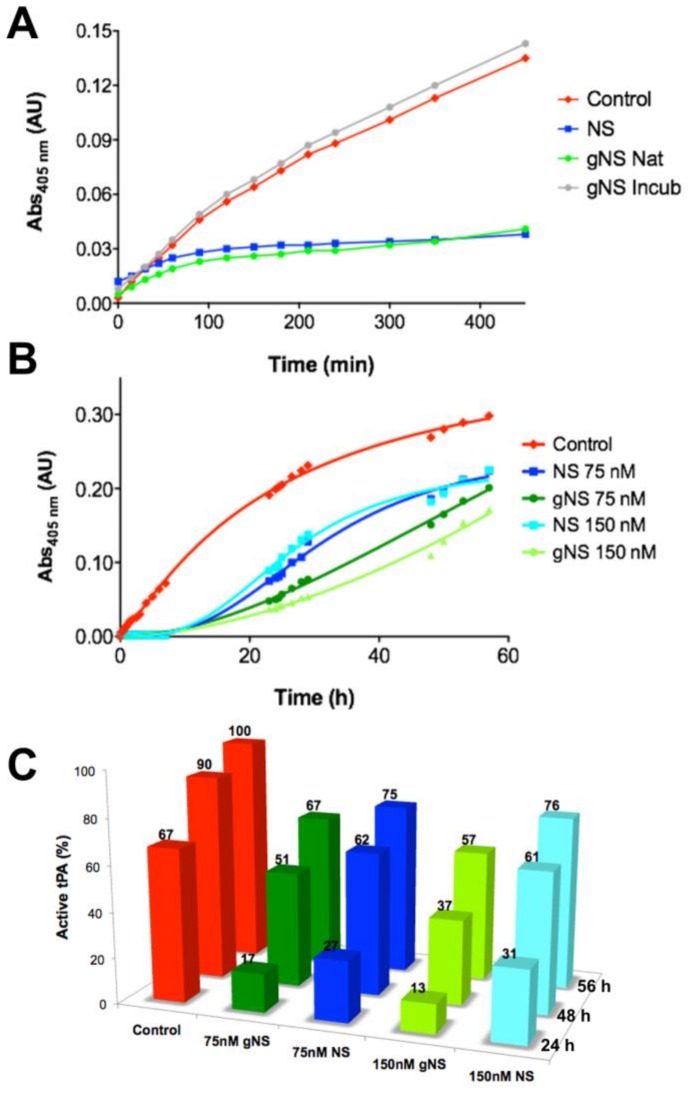
Comparison of tissue plasminogen activator (tPA) inhibition by NS and gNS. (**A**) tPA activity tests in the absence (red) and presence of 60 nM of NS (blue), native (green) or incubated for 24 h at 45 °C (grey) gNS. (**B**) The ability of gNS and NS to inhibit tPA at two different concentrations (75 and 150 nM) was compared. (**C**) The inhibitory efficiency of gNS or NS is reported in comparison to the control reaction. The activity of tPA in the positive control after 56 h is set to 100% and all other values have been normalised accordingly. In the control reaction, no gNS or NS was added.

Then the ability of gNS to inhibit tPA in vitro was assessed. The activity of tPA on its chromogenic substrate IPR-pNA was measured in the presence or absence of gNS or NS, monitoring absorbance at 405 nm. While in the absence of NS or gNS the absorbance due to IPR-pNA hydrolysis rapidly reached a plateau, in the presence of gNS and NS, the absorbance at 405 nm increased more slowly and remained almost constant, indicating that both NS and gNS displayed similar inhibitory activity against tPA (Figure 5A). The inhibition of tPA by a sample of gNS incubated for 24 h at 45 °C was also tested and tPA was completely active when compared to the control reaction (Figure 5A), indicating that, during incubation, gNS has lost its inhibitory activity. This is likely due to the formation of the inactive polymer and latent forms and the concomitant disappearance of native gNS.

The inhibition of tPA by gNS and NS was tested for longer times and at different concentrations (Figure 5B,C). It is apparent that gNS is a marginally more efficient inhibitor of tPA proteolytic activity since the substrate was hydrolysed more slowly in the presence of gNS than in the presence of NS (Figure 5B). Figure 5C shows more quantitively than panel 5B the different tPA inhibitions by NS and gNS. The percentage of active tPA at different times of incubation is reported in comparison with the control reaction without inhibitors. During the first 24 h of incubation time, the comparison of tPA activity in the presence of gNS or NS shows marked differences at both concentrations tested. In particular, curves in Figure 5B show that, after about 15 h, substrate hydrolysis by tPA is markedly increasing in the presence of NS, while, in the presence of gNS, tPA activity grows more slowly, displaying lower rate of hydrolysis for the first 50 h. At the end of the experiment (56 h), the difference in inhibitory efficiency tends to level up even though gNS was still a marginally better inhibitor than NS (Figure 5B,C). These observations strongly suggest a higher stability—i.e., slower rate of hydrolysis—of the gNS-tPA covalent complex compared to the one formed by NS-tPA. Although the gNS-tPA complex remains transient, the presence of the two N-glycans seem to stabilise the tPA-gNS complex with a consequent slower deacylation rate [14]. The full description of this observation and the molecular bases of this stabilisation by the presence of the glyco-antennas will require further characterisation in a future work.

### 2.5. Glycosylation Reduces Heat-Induced Polymerisation of gNS

**Figure 6:**
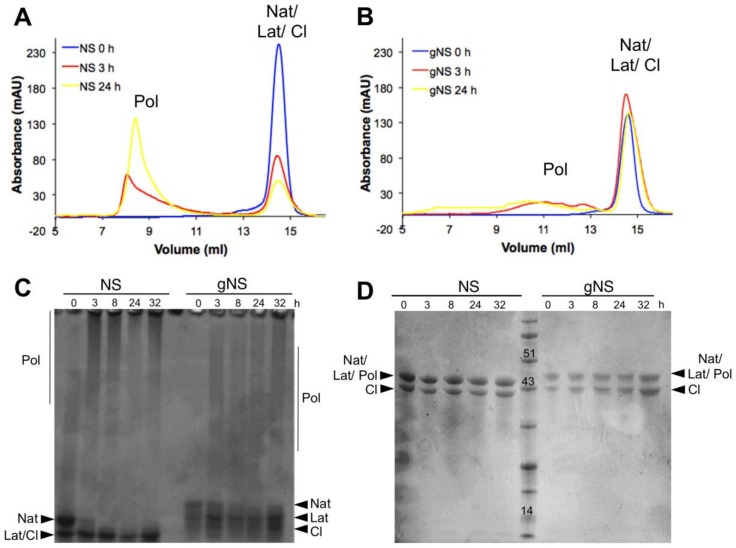
Heat-induced polymerisation of gNS. A solution of gNS or NS was incubated at 45 °C and analysed at different time points. SEC analysis of NS (**A**) or gNS (**B**) using an Increase Superdex 200 column. Aliquots of NS or gNS were analysed by non-denaturing (**C**) and denaturing (**D**) PAGE. Abbreviations: Nat: native; Cl: cleaved; Lat: latent; Pol: polymers.

The role of glycosylation during the heat-induced polymerization process was next evaluated. Solutions of gNS and NS at a concentration of 63.8 μM were incubated at 45 °C and the presence of polymeric species was assessed at different time points using SEC and both non-denaturing and denaturing PAGE (Figure 6). Most of the bacterially purified NS was already assembled into large polymeric species after 3 h of incubation and only a minor peak corresponding to monomeric NS was visible (Figure 6A). Non-denaturing PAGE confirmed the presence of very large polymers, as they were barely entering in the non-denaturating PAGE (Figure 6C). The denaturing PAGE analysis of aliquots of the same samples assessed the SDS-solubility of these species (Figure 6D). Thus, as previously shown [24], at 45 °C, NS undergoes rapid conformational changes, leading to the formation of a great excess of polymers over the latent conformer. 

In contrast, gNS displayed a remarkably different behaviour. First, even after 24 h of incubation, only a minor amount of polymeric form was detected by SEC (Figure 4B) and non-denaturing PAGE (Figure 4C). Moreover, polymers formed by gNS were eluted at 10–12 mL in SEC, in contrast to the large polymers formed by bacterially purified NS that were eluted into the dead volume of the column (Figure 6B). Analogously, in non-denaturating PAGE, gNS polymers displayed an apparent lower mass range (Figure 6C) compared to polymers formed by bacterially expressed NS. When analysed by denaturing PAGE, the gNS polymers dissolved and migrated as monomeric gNS, as observed for the bacterially purified counterpart (Figure 6D). It should be noted that, even though a small amount of polymers was observed upon incubation at 45 °C, most of the gNS remained monomeric, as shown in SEC and non-denaturating gel (Figure 6B,C). The different migration of gNS in the non-denaturating gel suggests that the native conformer disappears over time (Figure 6C). Overall, these data indicate that, in the presence of glycosylation, polymer formation is greatly reduced. Importantly, gNS polymerisation propensity is in line with previous observations in vivo and in cell models of FENIB where wild-type NS does not accumulate in polymeric deposits [26,37]. These results reinforce the hypothesis that the presence of glycan is crucial in preventing aberrant interaction between NS monomers [30].

The lack of inhibitory activity observed in vitro by incubated gNS (Figure 5A) suggests that the native gNS undergoes a conformational change resulting in the formation of the inactive latent conformation, as also suggested by Figure 6C. 

## 3. Materials and Methods

### 3.1. LEXSY Plasmid and Strain

The cassette containing the sequence of the human NS gene optimized for *Leishmainia tarentolae* codon bias, in fusion at the N-terminus with the sequence for the secretion signal peptide (5′-ATGGCTAGCCGTCTCGTCCGGGTGCTCGCAGCGGCAATGCTGGTGGCAGCCGCTGTCTCCGTCGCAATGGCC-3′) and the 6-His tag, was sub-cloned into the pLEXSY-sat2 plasmid (Jena Bioscience, Jena, Germany) using *NcoI* and *Kdel* restriction enzymes (New England Lab, NEB, Ipswich, MA, USA). pCloneGZ/NS and pLEXSY-sat2 were digested with *NcoI* and *Kdel*. The digestion products were gel-purified and ligated using the Quik Ligation kit (NEB). The resulting pLEXSY-sat2/NS plasmid was linearized with SwaI restriction enzyme (Thermo Fisher Scientific, Waltham, MA, USA). All the amplification steps were performed in *E. coli* XL1 cells (Invitrogen, Thermo Fisher Scientific, Waltham, MA, USA) at 30 °C for plasmid stability reasons. LEXSY host P-10 cells (Jena Biosciences) were transfected with linearized pLEXSY-sat2/NS plasmid and plated on freshly prepared agar plates (40 mM Hepes pH 7.4, 26 g/L brain heart infusion, 10% inactivated fetal calf serum, 1% bactoagar, 50 µg/mL hemin, 1 unit/mL penicillin and 0.1 mg/mL streptomycin, 100 µg/mL nourseothricin, NTC). After 2 weeks at 26 °C, colonies were inoculated into Brain Heart Infusion (BHI) medium supplemented with 50 µg/mL hemin, 1 unit/mL penicillin, 100 µg/mL streptomycin (BHI-complete medium) and 100 µg/mL NTC. 

Protein expression was evaluated by dot-blot using monoclonal anti-NS(m19) primary antibody and HRP-coupled anti-rabbit IgG. Membranes were developed using Pierce ECL Western Blotting substrate (Thermo Fisher Scientific, Waltham, MA, USA). All chemicals were purchased from Sigma Aldrich (St. Louise, MO, USA), and NTC was bought from Jena Bioscience.

### 3.2. gNS Expression and Purification

Transformed LEXSY cells were cultured at 26 °C in BHI complete medium, always added with NTC, in a ventilated tissue flask in static suspension in the dark. Cultures were split two times a week with a 1:20 dilution into fresh medium. For large-scale protein expression, cells were cultured 50 h under 220 rpm shaking at 26 °C in the dark and then collected by centrifugation during 15 min at 4500 *g*. The cell pellet was resuspended in the same volume of fresh BHI-complete medium and incubated for 16 h at 18 °C. Finally, cells were collected 15 min at 10,500 *g* and the supernatant was concentrated using an Amicon Millipore ultrafiltration system. Concentrated culture medium was dialyzed against buffer A (20 mM Tris-HCl pH 8.0, 10 mM imidazole, 300 mM NaCl) to remove hemin and loaded onto a 5-mL HiTrap Chelating HP Amersham column (GE Healthcare, Chicago, IL, USA) previously equilibrated with the same buffer. Proteins were eluted in buffer B (20 mM Tris-HCl pH 8.0, 250 mM imidazole, 300 mM NaCl) after a washing step at 10% of buffer B. Monomers were further isolated by gel filtration using an Hi Load 16/60 Superdex200 column (GE Healthcare) equilibrated in 10 mM Tris-HCl, 50 mM KCl, pH 8.0. All purifications steps were performed at 4 °C. All chemicals were purchased from Sigma-Aldrich, unless differently specified. NS concentration was measured by optical absorption using an extinction coefficient at 280 nm of 37,360 M^−1^ cm^−1^. Due to the impossibility to obtain a completely native sample of gNS, all experiments comparing gNS and bacterially expressed NS were performed using a mix of native and cleaved NS in a ratio similar to gNS samples allowing reliable comparisons.

### 3.3. NS Expression and Purification

*E. coli* BL21 Rosetta (DE3) pLysS Competent Cells Novagen (Merck KGaA) transformed with pQE81L plasmid carrying the human NS gene with a N-terminus 6-His tag were used to express and purify NS as reported in [6]. Briefly, bacterial cells were grown in Superior Broth (Molecular Dimension) containing 100 µg/mL ampicillin, and protein expression was induced with 0.2 mM isopropyl-β-D-thiogalactopyranoside at 17 °C for 16 h. NS was purified from the soluble fraction. Buffer A supplemented with cOmplete protease inhibitor cocktail (Hoffmann-La Roche, Basel, Switzerland) was added, and the cell pellets were immediately sonicated. Crude extract was clarified by centrifugation and filtered before being loaded onto a 1-mL HiTrap Chelating HP column (GE Healthcare) previously equilibrated with buffer A. Protein was eluted in 100% buffer B. Monomers were further isolated by gel filtration using an Hi Load 16/60 Superdex200 column (GE Healthcare) equilibrated in 10 mM Tris-HCl, 50 mM KCl, pH 8.0. All purification steps were performed at 4 °C and all chemicals were purchased from Sigma-Aldrich, unless differently specified. NS concentration was measured by optical absorption using an extinction coefficient at 280 nm of 37,360 cm^−1^ M^−1^. 

### 3.4. NS Autoproteolysis Assay

A solution of 63.8 µM of NS in 10 mM Tris-HCl, 50 mM KCl, pH 8 or 7.6 was incubated at 26 °C or 37 °C. At different time of incubation, an aliquot was subjected to denaturing-PAGE. Gels were stained with Coomassie Brilliant Blue R-250. The experiment was repeated at least three times, and representative gel is reported.

### 3.5. Deglycosylation Assay

Deglycosylation was performed using a peptide-N Glycosydase F (PNGase) deglycosylation kit (NEB) in non-denaturing conditions or an endoglycosydase H (EndoH) deglycosylation kit (NEB). According to the manufacturer’s instruction, 10 µg of gNS was diluted in GlycoBuffer and 2 µl of PNGaseF was added. Protein mix was incubated at 37 °C for 16 h before SDS-PAGE analysis. For EndoH digestion, 10 µg of gNS were diluted in Glycoprotein denaturing buffer and incubated 10 min at 80 °C prior to the addition of 2 µL of the enzyme. The mixture was incubated 5 h at 37 °C and analysed by SDS-PAGE. In both assays, a solution of 10 µg gNS were prepared in the same experimental conditions but with no enzyme as a negative control. The experiment was repeated at least three times and representative gels are reported.

### 3.6. MALDI-TOF MS Analysis

Matrix-assisted laser desorption ionisation-time of flight (MALDI-TOF) mass analysis was carried out on concentrated NS or gNS solutions. NS and gNS constructs have slightly different Histag constructs: the theoretical mass is 46,271 and 45,810 Da, respectively. Purified protein samples were diluted with 0.1% trifluoroacetic acid in water (TFA) to a final concentration of 10 pmol/µL, prior protein precipitation with 50% trichloroacetic acid (TCA). Each sample was loaded onto a MALDI target plate using the dry droplet technique and sinapinic acid (SA) in 0.1% TCA:acetonitrile (2:1) as matrix. Mass spectrometry analysis was carried out by a Bruker Daltonics Reflex III instrument (Bruker Daltonics, Billerica, MA, USA) equipped with a nitrogen laser (337 nm), operating in linear positive mode. Each spectrum was accumulated for at least 200 laser shots and protein calibration standards II (Bruker Daltonics) were used for calibration [38]. The PeptideMass tool (http://www.expasy.org/tools/peptide-mass.html) was used to calculate the theoretical masses of native and cleaved NS and gNS proteins and for identifying NS cleavage site. 

### 3.7. UHPLC-MS/MS Analysis 

Ultra performance liquid chromatography-tandem mass spectrometry (UHPLC-MS/MS) analysis were performed on gNS and deglycosylated tryptic peptides obtained by a standard in-gel digestion protocol [39] applied on gel bands of gNS and gNS deglycosylated with PNGaseF. After digestion, the peptides were desalted by C18 Ziptips (Millipore, Burlington, MA, USA) and injected on an Orbitrap Fusion mass spectrometer equipped with a nano-UHPLC system EASY 1000 (Thermofisher). Peptides were separated by a 1-h gradient (aqueous phase: milliQ water, 0.1% formic acid; organic phase: 80% acetonitrile, 20% milliQ water, 0.1% formic acid), detected by the orbitrap analyzer, and subjected to a first fragmentation step by Higher energy Collision Dissociation (HCD). The corresponding HCD fragmentation spectra allowed the for identification of unmodified peptides by the software Proteome Discoverer (Thermo Fisher). In the case of glycopeptides, instead, HCD fragmentation mainly generates intense reporter ions of the glyco-antenna, e.g., HexNAc, 204.09 Da. This feature was exploited to selectively trigger a second fragmentation step by EThcD, a combination of HCD and Electron Transfer Dissociation (ETD), in order to better identify the peptide sequence and map the modification site [40].

### 3.8. Circular Dichroism Analysis

CD measurements were carried out on 4.5 μM NS or gNS in 10 mM Tris-HCl, 50 mM KCl, pH 8.0, by a J-810 spectropolarimeter (Jasco, Mary’s Court Easton, MD, USA) equipped with a PFD-425S temperature controller module (Jasco), in a 0.1 cm path-length cuvette. Temperature ramps ware performed recording ellipticity at 216 nm during sample heating from 20 to 95 °C and following cooling from 95 to 20°C (temperature slope 1.0 °C/min). First, the derivative of the data has been calculated and plotted to determine the temperature of transitions. 

### 3.9. Intrinsic Protein Fluorescence Assay

The intrinsic tryptophan fluorescence of 11 µM monomeric or polymeric gNS was measured in a FP-8200 spectro-fluorimeter (Jasco) at 20 °C. Emission spectra from 310 to 410 nm were recorded and 295 nm was used as an excitation wavelength. Each trace is the average of the accumulation of three spectra.

### 3.10. Polymerisation Assay 

Solution of 63.8 μM NS or gNS in 10 mM Tris-HCl pH 8.0 and 50 mM KCl were incubated at 45 °C. Aliquots at different incubation time were collected and analysed by size exclusion chromatography (SEC) using a Superdex 200 Increase 10/300 GL column (GE Healthcare Europe GmbH). An aliquot of each sample was subsequently subjected to non-denaturating polyacrylamide gel electrophoresis. The experiment was repeated at least three times and representative gels are reported.

### 3.11. Non-Denaturating Polyacrylamide Gel Electrophoresis (PAGE)

Samples were prepared as previously reported [41]. Briefly, aliquots of gNS or NS at different times of incubation at 45 °C were collected and mixed at a 1:1 volume ratio with a non-denaturing loading buffer (250mM Tris-HCl, 50% glycerol, 0.5% bromophenol blue, pH 6.8). Samples were separated into 7.5 % non-denaturating polyacrylamide gels run at 90 V for 2 h at 4 °C to prevent sample denaturation and/or polymer dissociation. Gels were stained with Coomassie Brilliant Blue R-250. The experiment was repeated at least three times and representative gels are reported.

### 3.12. Inhibitory Activity Test

The inhibition of two-chain tissue plasminogen activator (2ctPA, American Diagnostica, Pfungstadt, Germany) by NS or gNS was determined in the presence of the chromogenic substrate H-D-Ile-Pro-Arg-p-nitroanilide (IPR-pNA; Chromogenix, Werfer, L’Hospitalet de Llobregat, Spain) by recording the pNA accumulation upon substrate cleavage. The reaction was prepared mixing 60, 75 or 150 nM NS, 250 μM IPR-pNA, 1 nM 2ctPA in 50 mM Tris-HCl pH 7.4, 10 mM Na_2_HPO_4_, 150 mM NaCl, and 0.1% Tween and incubated at 25 °C [42]. The reaction was monitored at 405 nm in an Ultraspec 2100 Pro spectrophotometer (Amersham Bio, Little Chalftont, UK). The curves shown are representative of all the performed experiments. 

## 4. Conclusions

In summary, we report here the expression, purification and characterisation of human N-glycosylated NS. Recombinant gNS is properly glycosylated in the physiologic positions N154 and N321, which were previously identified [30]. Moreover, gNS is properly folded and it is a marginally better in vitro inhibitor of tPA activity than non-glycosylated NS. Crucially, the presence of the two N-glycosylation chains impacts gNS propensity to pathologic polymerisation. More specifically, while non-glycosylated NS efficiently forms polymers in vitro, the formation of gNS polymers is slow and marginal. Although the present data do not provide a detailed explanation for this observation, gNS low polymerisation propensity may be due to the steric hindrance of the two glycan chains and to the slight increase in gNS fold stability, as seen in Figure 4B,C. Here, we show that the artifactual polymerisation propensity observed for wild-type NS expressed in bacteria is due to the absence of a proper glycosylation, while gNS displays a behaviour closely resembling what has been observed in vivo and in cell systems. Thus, gNS may be seen as a valuable tool to properly characterise the polymerisation propensity of the FENIB mutants in vitro.

## Supplementary Materials

Supplementary materials can be found at https://www.mdpi.com/1422-0067/21/9/3235/s1.

## Abbreviations

AC	Affinity chromatography
CD	Circular dichroism
Cl	Cleaved neuroserpin
EndoH	Endoglycosidase H
FENIB	Familial encephalopathy with neuroserpin inclusion bodies
Frag	Proteolytic neuroserpin fragment
gNS	Glycosylated NS
Lat	Latent neuroserpin
LEXSY	Leishmania expression system
MALDI-TOF MS	Matrix Assisted Laser Desorption/Ionization - Time of Flight Mass Spectrometry
Nat	Native neuroserpin
NS	Neuroserpin
PNGaseF	Peptide -N-Glycosidase F
Pol	Polymeric neuroserpin
RCL	Reactive centre loop
SDS-PAGE	Sodium Dodecyl Sulphate - PolyAcrylamide Gel Electrophoresis
SEC	Size exclusion chromatography
SERPIN	Serine Protease Inhibitor
tPA	Tissue Plasminogen Activator
UHPLC MS/MS	Ultra high performance liquid chromatography - tandem mass spectrometer
