# Supplementary material for: Glycosylation Tunes Neuroserpin Physiological and Pathological Properties
Source: Int J Mol Sci. 2020 May 3;21(9):3235. doi: 10.3390/ijms21093235 (PMC7247563; doi:10.3390/ijms21093235)
Supplement: Supplementary file 1 [file ijms-21-03235-s001.pdf]

International Journal of
Molecular Sciences

MDPI

Supplementary material

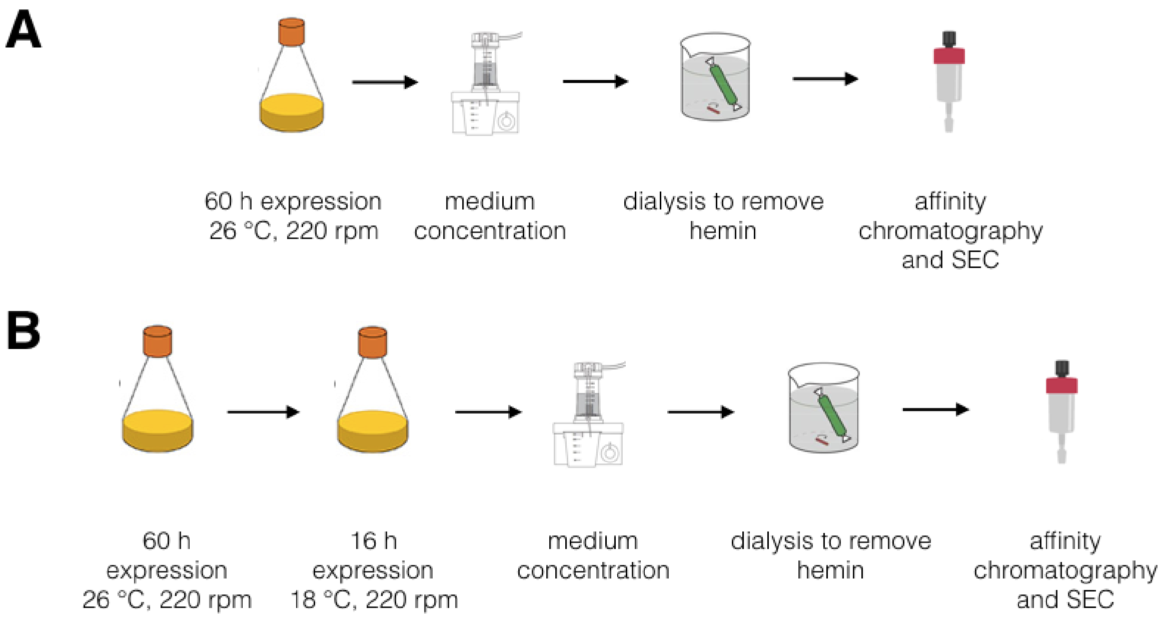

Figure S1. Schematic representation of expression and purification protocol of gNS using LEXSY. (A) Schematic representation of the initial protocol followed to express and purify gNS. (B) Schematic representation of the optimized protocol followed to express and purify gNS.

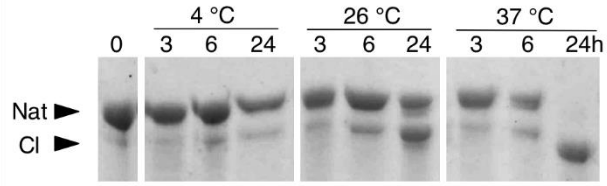

Figure S2. Monitoring NS spontaneous proteolysis at different temperature. Denaturing-PAGE analysis of 63.8 μM NS in 10 mM Tris-HCl, 50 mM KCl, pH 8.0 incubated at 4, 26 or 37 °C at different incubation time.

Table 1. gNS peptides identified by UHPLC-MS/MS. Peptide position is numbered according to UniProt (entry Q99574); SP: signal peptide used in this work. L: peptide length. Mox: methionine oxidation; HexNAc: N-acetylhexoseamine; when present, numbers into brackets indicate the identified position of the post-translational modification (PTM) on the peptide. MC: number of missed-cleaved sites during trypsin digestion. CS: charge state of the identified peptide. m/z: mass-to-charge ratio of the identified peptide. RT: retention time (min).

Position	L	Sequence	PTMs	MC	CS	m/z	RT
SP, 17-36	31	GSHHHHHHTDPTGATFPEEAIADLSVNMYNR	1 Mox [28]	0	5+	699,72	17,4
37-70	34	LRATGEDENILFSPLSIALAMGMMELGAQGSTQK	-	1	3+	1193,93	31,4
37-70	34	LRATGEDENILFSPLSIALAMGMMELGAQGSTQK	1 Mox	1	3+	1199,26	32,0
37-70	34	LRATGEDENILFSPLSIALAMGMMELGAQGSTQK	2 Mox [23]	1	3+	1204,60	37,6
37-70	34	LRATGEDENILFSPLSIALAMGMMELGAQGSTQK	3 Mox [21, 23, 24]	1	4+	907,70	33,7
39-70	32	ATGEDENILFSPLSIALAMGMMELGAQGSTQK	-	0	3+	1104,20	35,0
39-70	32	ATGEDENILFSPLSIALAMGMMELGAQGSTQK	1 Mox	0	3+	1109,53	35,9
39-70	32	ATGEDENILFSPLSIALAMGMMELGAQGSTQK	2 Mox [21]	0	3+	1114,87	37,3
39-70	32	ATGEDENILFSPLSIALAMGMMELGAQGSTQK	3 Mox [19, 21, 22]	0	3+	1120,20	36,5
74-82	9	HSMGYDSLK	-	0	2+	519,24	7,1
74-82	9	HSMGYDSLK	1 Mox [3]	0	2+	527,24	2,6
74-91	18	HSMGYDSLKNGEEFSFLK	-	1	3+	697,00	20,9
74-91	18	HSMGYDSLKNGEEFSFLK	1 Mox [3]	1	2+	1052,99	19,2
83-91	9	NGEEFSFLK	-	0	2+	535,76	18,1
83-100	18	NGEEFSFLKEFSNMVTAK	1 Mox [14]	1	3+	698,67	27,1
92-107	16	EFSNMVTAKESQYVMK	2 Mox [5, 15]	1	3+	641,97	12,1
108-129	22	IANSLFVQNGFHVNEEFLQMMK	1 Mox	0	3+	871,42	27,6
108-129	22	IANSLFVQNGFHVNEEFLQMMK	2 Mox [20, 21]	0	3+	876,76	26,0
108-130	23	IANSLFVQNGFHVNEEFLQMMKK	1 Mox	1	4+	685,85	25,0
108-130	23	IANSLFVQNGFHVNEEFLQMMKK	2 Mox [20, 21]	1	4+	689,84	23,0
130-153	24	KYFNAAVNHVDFSQNVAVANYINK	-	1	4+	682,35	21,8
131-152	23	YFNAAVNHVDFSQNVAVANYINK	-	0	3+	866,76	29,9
**154-164**	**11**	**WVENNTNNLVK**	**1 HexNAc [4]**	**0**	**2+**	**767,38**	**12,8**
171-187	17	DFDAATYLALINAVYFK	-	0	2+	968,00	42,8
188-200	13	GNWKSQFRPENTR	-	1	4+	405,71	8,3
192-200	9	SQFRPENTR	-	0	3+	378,86	3,5
260-272	13	QEVPLATLEPLVK	-	0	3+	479,62	25,2
260-285	26	QEVPLATLEPLVKAQLVEEWANSVKK	-	2	4+	730,66	31,7
273-284	12	AQLVEEWANSVK	-	0	2+	687,36	18,1
273-285	13	AQLVEEWANSVKK	-	1	3+	501,27	13,7
285-294	10	KQKVEVYLPR	-	2	3+	420,59	8,5
286-294	9	QKVEVYLPR	-	1	3+	377,89	11,2
295-304	10	FTVEQEIDLK	-	0	2+	611,32	21,0
295-308	14	FTVEQEIDLKDVLK	-	1	2+	838,96	30,0
**319-335**	**17**	**DANLTGLSDNKEIFLSK**	**1 HexNAc(2) [3]**	**1**	**3+**	**757,71**	**21,2**
340-362	23	SFLEVNEEGSEAAAVSGMIAISR	1 Mox [18]	0	2+	1192,08	46,5
385-393	9	TGTILFMGR	-	0	2+	498,27	19,2
394-410	17	VMHPETMNTSGHDFEEL	2 Mox [2, 7]	0	3+	669,28	13,2
